# Simultaneous Pneumothorax and Pulmonary Embolism With Concurrent COVID-19 After Shoulder Arthroscopy: A Case Report

**DOI:** 10.7759/cureus.95010

**Published:** 2025-10-20

**Authors:** Kazuhiro Ikeda, Shotaro Teruya, Hiromitsu Tsuge, Takeshi Makihara, Shinzo Onishi

**Affiliations:** 1 Institute of Medicine, Department of Orthopedic Surgery, University of Tsukuba, Tsukuba, JPN; 2 Department of Orthopedic Surgery, Kikkoman General Hospital, Noda, JPN; 3 Department of Orthopedic Surgery, Kasumigaura Medical Center, Tsuchiura, JPN

**Keywords:** complication, covid-19, pneumothorax, pulmonary embolism, shoulder arthroscopy

## Abstract

Shoulder arthroscopy is generally safe; however, pneumothorax and pulmonary embolism can rarely occur, and their coexistence poses substantial diagnostic challenges. We report a case in which both developed after arthroscopic surgery, together with concurrent COVID-19 infection.

A 72-year-old woman sustained an anterior shoulder dislocation with rotator cuff tears and a large bony Bankart lesion. She underwent arthroscopic Bankart and cuff repair 20 days after injury. On postoperative day (POD) 2, a pneumothorax was detected and treated with chest drainage; however, hypoxemia persisted. Contrast-enhanced CT on POD3 revealed bilateral pulmonary embolism. On POD6, she developed sore throat and fever and was diagnosed with COVID-19. Anticoagulation stabilized her condition, and she was discharged on POD27.

This case underscores three lessons: (1) avoid anchoring on the first diagnosis because pneumothorax and pulmonary embolism share symptoms; persistent hypoxemia after drainage warrants early Contrast-enhanced CT; (2) when high-risk complications coexist, management must explicitly address bidirectional treatment interactions; and (3) minimizing operative time is a key preventive measure, and in elderly patients with large glenoid defects and cuff deficiency, primary reverse shoulder arthroplasty may be a reasonable alternative to complex arthroscopic reconstruction.

## Introduction

Shoulder arthroscopy is generally considered minimally invasive with a low adverse-event burden; the 90-day overall complication rate is about 1.2% [1]. Among these events, pneumothorax and pulmonary embolism are rare yet potentially fatal [2-5]. Pneumothorax occurs in ≤0.2% of cases and is considered a procedure-specific complication [6]. Meanwhile, venous thromboembolism occurs in roughly 0.24% of patients after shoulder arthroscopy, with pulmonary embolism reported in 0.01-0.06% [7]; the lateral decubitus position may further increase risk [5]. Both conditions require rapid diagnosis and prompt initial management [8,9]. However, concomitant pneumothorax and pulmonary embolism make both diagnosis and management difficult [10]. We encountered an exceptionally rare case in which both conditions developed after shoulder arthroscopy in combination with COVID-19 infection. The overlap of pneumothorax and pulmonary embolism makes the differential diagnosis of postoperative respiratory and circulatory disturbances particularly difficult, while the coexistence of COVID-19 added further challenges to hospital infection control in the context of chest drainage and anticoagulation therapy. This case is reported for its rarity and for the insights it provides regarding the overlapping presentation and the complexities of management.

## Case presentation

**Figure 1:**
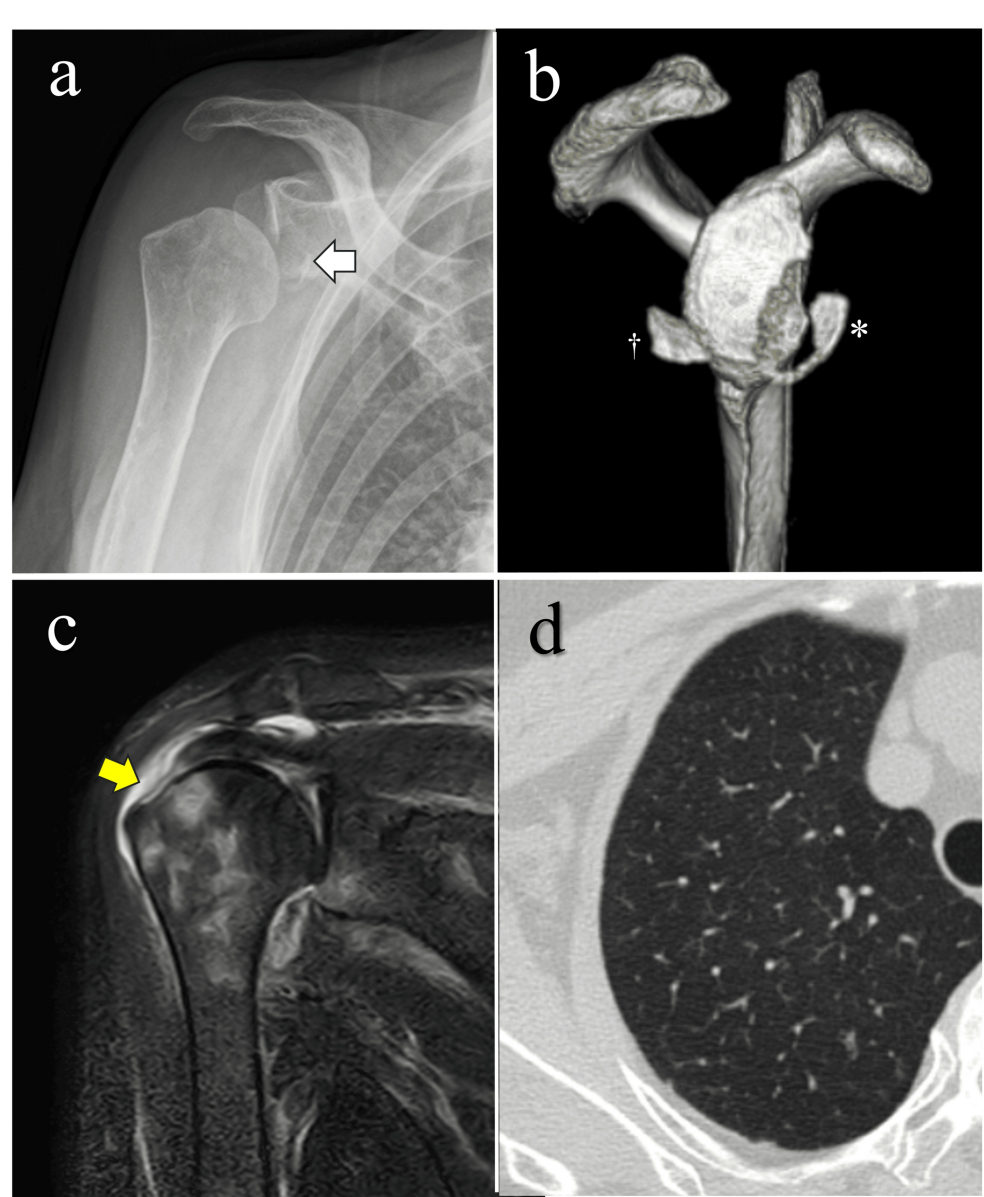
Preoperative imaging findings (a) Plain radiograph showing a glenoid bone defect (arrow) with poor joint congruency. (b) Three-dimensional computed tomography (CT) demonstrating a large bony Bankart lesion (asterisk) and a glenoid bone fragment (dagger). (c) Magnetic resonance imaging (MRI) revealing a full-thickness tear of the supraspinatus tendon (yellow arrow). (d) Chest CT showing no evidence of emphysematous changes.

A 72-year-old woman sustained a right anterior shoulder dislocation after a fall. Although closed reduction was performed at the initial hospital, she continued to experience shoulder pain and instability and was referred to our institution 10 days after injury (Table 1). Imaging findings revealed a large bony Bankart lesion involving 42% of the glenoid width and a full-thickness supraspinatus tendon tear (Figure 1). Preoperative laboratory tests revealed no coagulation abnormalities (Table 2). Based on these findings, arthroscopic Bankart repair (ABR) combined with arthroscopic rotator cuff repair (ARCR) was planned 20 days after injury.

**Table 1:** Patient characteristics

Item	Details
Chief complaint	Right shoulder pain and a sense of instability
Medical history	Diabetes mellitus and hypertension
Medications	Sitagliptin, amlodipine, and candesartan
Social history	No history of smoking
Body habitus	Height, 151 cm; weight, 58 kg; body mass index, 25.4

**Table 2:** Preoperative laboratory findings ALT, alanine aminotransferase; APTT, activated partial thromboplastin time; AST, aspartate aminotransferase; BUN, blood urea nitrogen; Cre, creatinine; CRP, C-reactive protein; Hb, hemoglobin; INR, international normalized ratio; Plt, platelet count; PT, prothrombin time; WBC, white blood cell count

Parameters	Patient Value	Reference Range	Unit
WBC	7100	4500-8000	/µL
Hb	12.2	12.0-16.0	g/dL
Plt	16.6	14.0-34.0	10⁴ /μL
PT	13.1	10.5-13.5	seconds
PT-INR	1.11	-	-
APTT	33.2	26.1-35.6	seconds
AST	16	7-38	U/L
ALT	12	4-43	U/L
BUN	12.0	8.0-20.0	mg/dL
Cre	0.65	0.36-1.06	mg/dL
CRP	0.1	0.0-0.3	mg/dL

Surgical procedure

**Figure 2:**
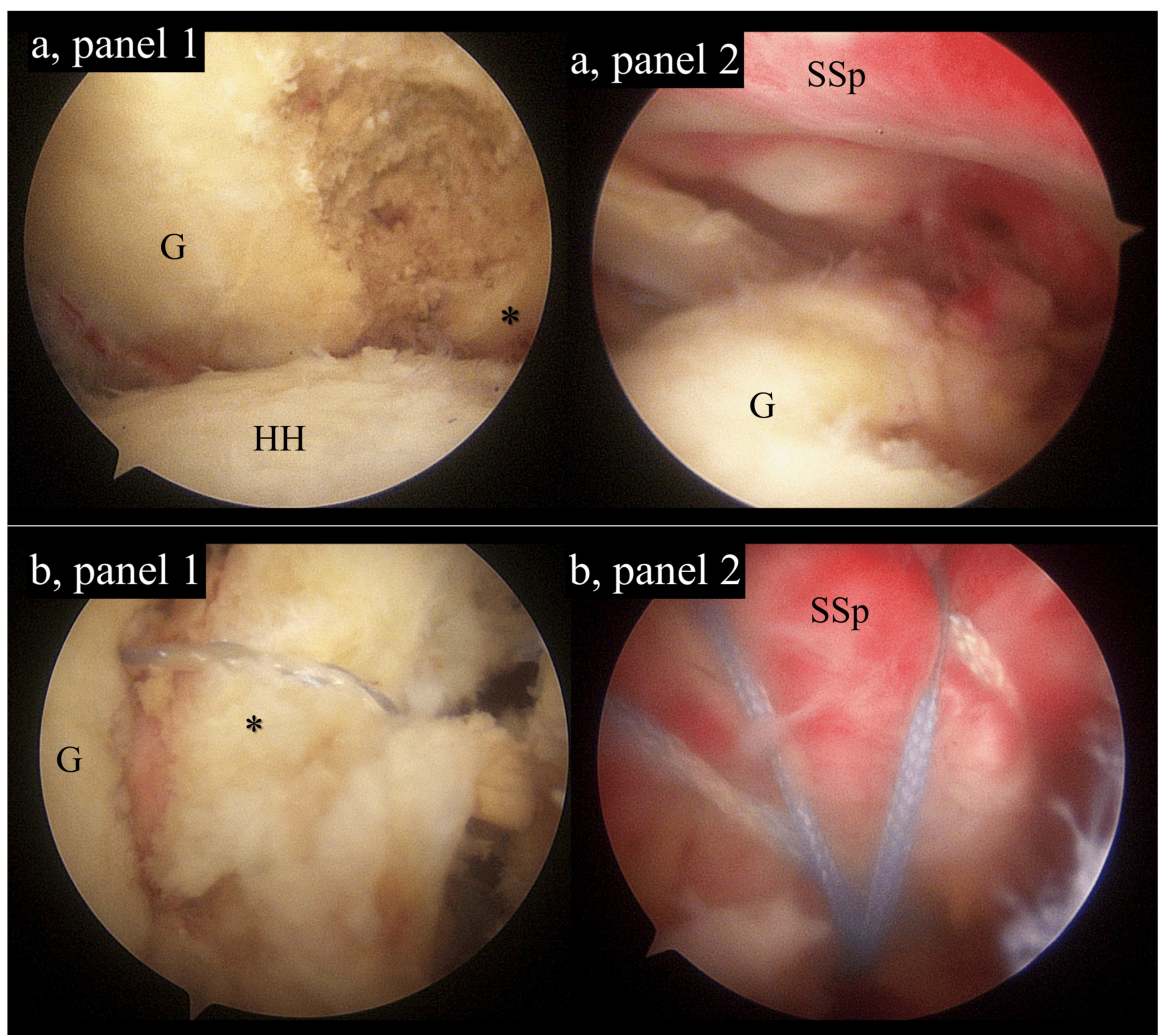
Arthroscopic findings (a) Before repair. (a, panel 1) Large bony Bankart lesion (asterisk) on the glenoid (G); osteoarthritic changes on the humeral head (HH). (a, panel 2) Full-thickness tear of the SSp. (b) After repair. (b, panel 1) Fixation of the bony fragment (asterisk) to the glenoid (G) with suture anchors. (b, panel 2) Bridging suture of the SSp. G, glenoid; HH, humeral head; SSp, supraspinatus tendon

Under general anesthesia in the beach-chair position, we performed surgery without regional block (Figure 2). For the bony Bankart lesion, three suture anchors were placed at the 1-, 3-, and 5-o’clock positions on the glenoid, and the fragment was fixed using a single-row technique. For the supraspinatus tendon tear, ARCR was performed with two medial anchors and two lateral knotless anchors in a bridging suture configuration. The operative time was four hours, and intraoperative blood loss was minimal. Irrigation was maintained with a pump at approximately 40 mmHg; mechanical prophylaxis consisted of intermittent pneumatic compression and elastic stockings applied from induction until ambulation; and no intraoperative complications occurred, with hemodynamic and respiratory status remaining stable throughout the procedure. 

Postoperative course

Postoperative day (POD) 1: She had no dyspnea and was able to walk independently. Her SpO₂ was 99% with 1 L oxygen via nasal cannula in the morning, while it decreased to the low 90s in the afternoon.

**Figure 3:**
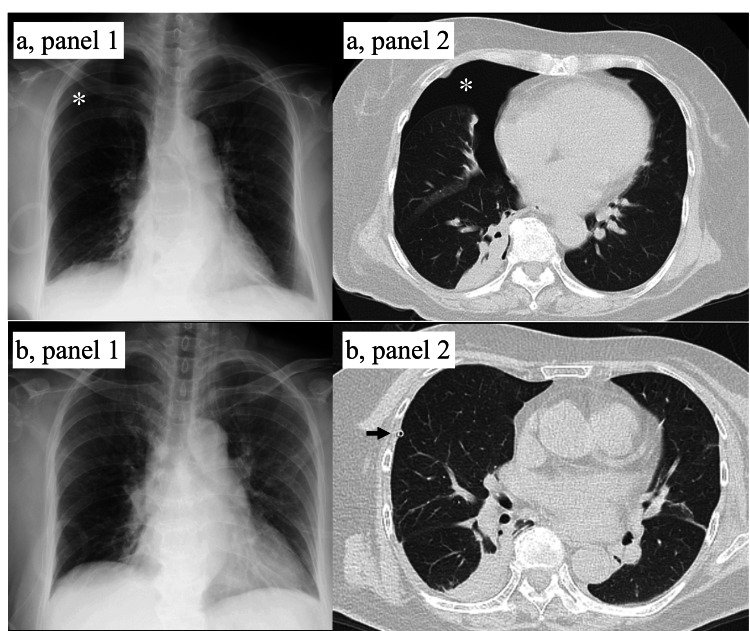
Radiological findings of pneumothorax (a) Before drainage. (a, panel 1) Chest radiograph on POD2 showing right pneumothorax (asterisk). (a, panel 2) Chest CT scan on POD2 confirming right pneumothorax (asterisk) without subcutaneous emphysema. (b) After chest tube drainage. (b, panel 1) Chest radiograph showing full lung expansion. (b, panel 2) Chest CT scan demonstrating re-expansion of the right lung (arrow: chest tube).

POD2: She had no subjective dyspnea, while she experienced right-sided chest pain on deep inspiration. Subsequently, SpO₂ dropped to the 80s. Chest radiography revealed a right pneumothorax, and a 10-Fr chest tube was inserted. Drainage promptly expanded the right lung, with no subsequent air leakage (Figure 3). Nevertheless, oxygenation did not improve, and 4 L oxygen supplementation was required. Arterial blood gas analysis showed pCO₂ 44.2 mmHg, pO₂ 72.0 mmHg, and HCO₃⁻ 26.6 mmol/L, indicating hypoxemia without hypercapnia. Therefore, we managed her conservatively with close observation on that day.

**Figure 4:**
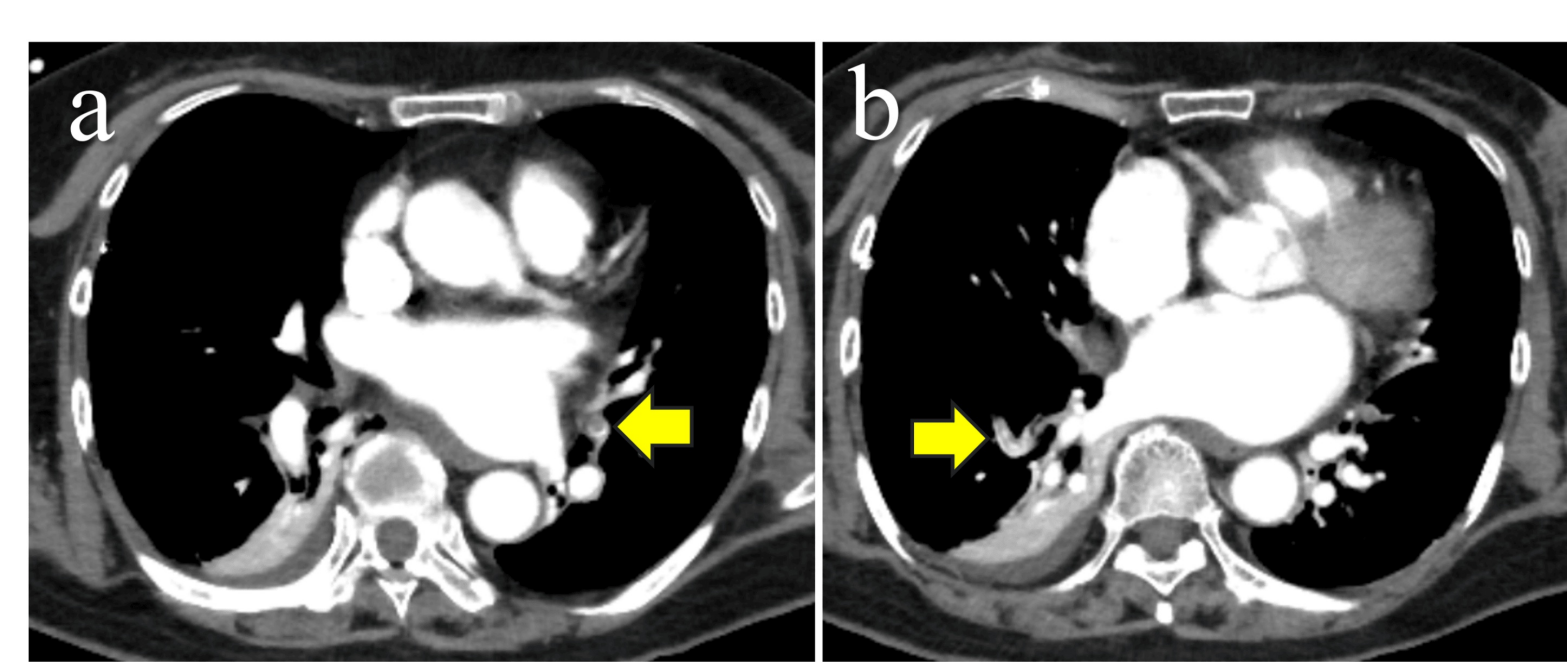
Contrast-enhanced CT findings of pulmonary embolism Multiple emboli in the pulmonary arteries of the right lung (a) and left lung (b) (arrows).

POD3: The patient exhibited persistent tachycardia (heart rate 143/minute) with unchanged oxygen demand (4 L via face mask). Blood tests showed an elevated D-dimer level of 4.5 μg/mL. Contrast-enhanced CT revealed multiple emboli in both pulmonary arteries and established the diagnosis of pulmonary embolism (Figure 4). There was no thrombus in the lower extremity veins. Anticoagulation with heparin (18,000 U/day), together with bed rest, elastic stockings, and a foot pump, was initiated.​​​​​​​

POD6: Low-grade fever (37.4°C) and pharyngeal discomfort appeared. Antigen testing confirmed COVID-19 infection (COV (1200) >10,000). Respiratory status was preserved (oxygen demand 1 L, SpO₂ 97%). Hospital policy required isolation for infection control.

**Figure 5:**
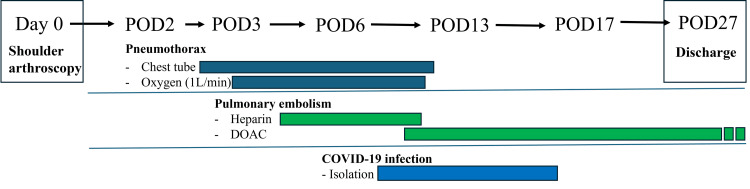
Timeline of postoperative clinical events DOAC, direct oral anticoagulant; POD, postoperative day

POD7-27: The patient started apixaban and gradually tapered heparin. The chest tube was removed on POD13. Both pulmonary embolism and pneumothorax thereafter progressed without further complications. She initiated gait training from POD15, terminated isolation on POD17, and was discharged on POD27 (Figure 5).

**Figure 6:**
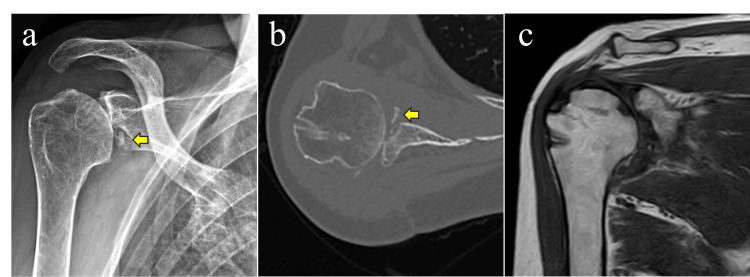
Imaging findings at one year postoperatively (a) Plain radiograph showing nonunion of the glenoid fragment (arrow) with articular incongruity. (b) Axial CT view demonstrating nonunion and fragmentation of the glenoid fragment (arrow). (c) MRI confirming rotator cuff healing.

Postoperative year (POY) 1: Forward elevation was limited to 60°, external rotation to 40°, and internal rotation reached only the buttock. Imaging findings demonstrated nonunion of the glenoid fragment, whereas the rotator cuff showed complete healing (Figure 6).

POY2: Range of motion remained unchanged, and pain was elicited during shoulder motion. Although reverse shoulder arthroplasty (RSA) was proposed as a salvage procedure, the patient declined surgery due to concerns about complications.

## Discussion

This patient developed three serious complications in close succession after shoulder arthroscopy-pneumothorax, pulmonary embolism, and COVID-19 infection - a constellation that is exceedingly rare. Each condition can be life-threatening on its own; when they coexist, the evaluation of postoperative respiratory and circulatory abnormalities becomes far more difficult [10]. A key diagnostic lesson in this case was the persistence of hypoxemia despite prompt resolution of the pneumothorax after chest drainage. Because pneumothorax and pulmonary embolism share clinical features such as dyspnea, pleuritic chest pain, hypoxemia, and tachycardia [8,9], anchoring on the first diagnosis risks missing the second. If oxygenation does not improve after effective drainage, clinicians should maintain a low threshold for contrast-enhanced CT to exclude pulmonary embolism.

Another consideration is potential pathophysiologic interplay. COVID-19-related hyperinflammation and coagulopathy have been associated with both spontaneous pneumothorax and acute pulmonary embolism [11,12]. In addition, pulmonary infarction secondary to pulmonary embolism can sometimes cause alveolar rupture, leading to a secondary pneumothorax [10]. By contrast, this case showed no persistent pleural communication after drainage, and COVID-19 was diagnosed four days later. These findings make a direct causal relationship unlikely. The two events were more likely driven by distinct but overlapping risk factors related to the patient, the surgical procedure, and the perioperative context.

Although pneumothorax after shoulder arthroscopy is very uncommon (<0.2%), it is recognized as a procedure-related complication [6]. Proposed mechanisms include mechanical effects on the thorax from patient positioning (lateral decubitus or beach-chair) and traction, pressure changes from irrigation and suction systems, and inadvertent air ingress through portals-particularly during extensive extra-capsular work in the subacromial space-with air dissecting along fascial planes into the pleura [4,6,13]. By contrast, beach-chair shoulder arthroscopy is not regarded as a specific risk factor for pulmonary embolism [14]. Our patient developed pulmonary embolism despite standard thromboprophylaxis, underscoring that rare thromboembolic events can still occur.

From a preventive standpoint, minimizing operative time is likely the most impactful modifiable measure [6]. In this case, combined glenoid reconstruction and rotator cuff repair resulted in a four-hour procedure. Given the patient’s age, a large glenoid bone loss, and cuff insufficiency, primary RSA would have been a reasonable alternative: it shortens operative duration, restores elevation without relying on cuff function, and provides stability through a semi-constrained implant [15]. Once severe postoperative complications occur, patients may hesitate to undergo salvage procedures. Surgical planning at the index operation should therefore integrate local pathology with systemic risk and the likelihood of future reoperation.

Management also required attention to therapeutic interactions. Anticoagulation in the presence of a chest tube increases the risk of hemothorax and drain-related complications [16]. Isolation for COVID-19 imposed resource and staffing constraints, which in turn influenced decisions regarding drain management. In our case, the chest tube showed no air leak from the outset. Despite this, removal was deferred for 11 days to prioritize safety under the constraints of infection control measures. When multiple high-risk complications coexist, a cautious, stepwise strategy that explicitly accounts for the bidirectional effects of each treatment modality is essential.

## Conclusions

This case highlights an exceptionally rare concurrence of pneumothorax, pulmonary embolism, and COVID-19 infection after shoulder arthroscopy, in which their coexistence made postoperative cardiopulmonary assessment and management particularly challenging.

Three key lessons emerge. First, pneumothorax and pulmonary embolism share overlapping symptoms, and anchoring bias must be avoided; persistent hypoxemia after chest drainage should prompt early contrast-enhanced CT. Second, when multiple complications coexist, management should be stepwise and cautious, explicitly accounting for the interactions between chest drainage, anticoagulation, and infection-control measures. Third, from a preventive perspective, minimizing operative time is essential, and in elderly patients with large glenoid fractures and cuff deficiency, primary RSA may be a reasonable alternative to complex arthroscopic reconstruction. In conclusion, optimizing safety in shoulder arthroscopy requires heightened awareness of these rare but potentially fatal complications, as well as prompt diagnosis, coordinated management, and prudent surgical decision-making.
